# Editorial for the Special Issue “Nanomaterials Ecotoxicity Evaluation”

**DOI:** 10.3390/nano13212878

**Published:** 2023-10-30

**Authors:** Xiaoshan Zhu, Jian Zhao

**Affiliations:** 1 College of Ecology and Environment, Hainan University, Haikou 570208, China; 2 College of Environmental Science and Engineering, Ocean University of China, Qingdao 266100, China; jzhao@ouc.edu.cn

Nanotechnology has made enormous progress over the last few decades, and the current use of nanomaterials is rapidly increasing. As a result, the continuous release of engineered nanomaterials (ENPs) into air, water, and soil has raised concerns about the possible adverse consequences for environmental and human health.

In this Special Issue, we aim to present a multidisciplinary overview of recent scientific articles that delve into various aspects of nanomaterials’ ecotoxicity evaluation associated with their occurrences, behavior, fate, and bioavailability. Such an evaluation is critical for scientists, legislators, business leaders, and the public to understand and develop effective solutions to the potential impacts of nanomaterials. Two reviews are also noteworthy. To address the risks of nanomaterials in the aquatic environment, Yuan et al. reviewed the environmental impact of nanomaterials with inorganic sunscreens [1]. For the risks of nanomaterials in the soil environment, Suazo-Hernández et al. reviewed the impact of ENPs on the physical and chemical properties of soils [2].

The toxicity of nanomaterials remains a significant issue. Lu et al. studied how low concentrations of silver nanoparticles induced novel toxic effects on aquatic ferns [3]. Environmental factors can also affect the environmental behavior of nanomaterials and change their toxic effects, for example, humic acid affects the adsorption and suspension behavior of carbon nanotubes [4].

The co-effect of nanomaterials and heavy metal pollutants remains a concern. On the one hand, the combined effect of nanomaterials and heavy metals leads to higher ecological risks. Zhou et al. studied CuO and Fe_3_O_4_ nanomaterials to enhance the bioaccumulation and toxicity of arsenic in marine mussels [5]. Similarly, Wang et al. found that CuO NPs promoted the phytotoxicity and accumulation of cadmium in vegetables [6]. On the other hand, the application of nanomaterials will reduce the accumulation of heavy metals in crops. Nano-Fe may modify the microbial community and decrease the soil-available Cd and As contents, inhibit the absorption of Cd and As by the roots, and decrease the transport of Cd and As in rice grains and the risk of intake in humans [7].

The effects of nanoplastics on organisms have attracted much attention. Wang et al. identified multiple mechanisms of toxicity on microalgae induced by nanoplastics [8]. The synergistic impact of microplastics and organic pollutants remains poorly understood in the marine environment. Zhou et al. implied that MPs in synergy with organic pollutants can be more harmful to marine organisms [9].

Nanopesticides are increasingly considered an emerging alternative due to their higher efficiency and lower environmental impacts. Although the public is not familiar with nanopesticides, they have positive attitudes toward their future development and support labeling nanoscale ingredients on products [10]. Ganilho et al. also assessed the environmental risks of lipid nanoparticles loaded with lambda-cyhalothrin [11].

To conclude, this Special Issue presents several examples of the latest advancements in assessing nanomaterials’ ecotoxicity. We hope readers will enjoy reading these articles and find them useful in their research.
